# Synergistic effects of novel penicillin-binding protein 1A amino acid substitutions contribute to high-level amoxicillin resistance of *Helicobacter pylori*

**DOI:** 10.1128/msphere.00089-24

**Published:** 2024-08-01

**Authors:** Alain Cimuanga-Mukanya, Evariste Tshibangu-Kabamba, Patrick de Jesus Ngoma Kisoko, Kartika Afrida Fauzia, Fabien Mbaya Tshibangu, Antoine Tshimpi Wola, Pascal Tshiamala Kashala, Dieudonné Mumba Ngoyi, Steve Ahuka-Mundeke, Gunturu Revathi, Ghislain Disashi-Tumba, Yasutoshi Kido, Takashi Matsumoto, Junko Akada, Yoshio Yamaoka

**Affiliations:** 1 Department of Environmental and Preventive Medicine, Faculty of Medicine, Oita University, Oita, Japan; 2 Department of Internal Medicine, Faculty of Medicine, Pharmacy and Public Health, University of Mbujimayi, Mbujimayi, Democratic Republic of Congo; 3 Department of Virology and Parasitology & Research Center for Infectious Disease Sciences, Graduate School of Medicine, Osaka Metropolitan University, Osaka, Japan; 4 Department of Internal Medicine, Faculty of Medicine, University of Kinshasa, Kinshasa, Democratic Republic of Congo; 5 Research Centre for Preclinical and Clinical Medicine, National Research and Innovation Agency, Cibinong Science Center, West Java, Indonesia; 6 Astrid Clinics, Gastroenterology and Hepatology Section, Kinshasa, Democratic Republic of Congo; 7 Department of Parasitology, National Institute of Biomedical Research (INRB), Kinshasa, Democratic Republic of Congo; 8 Department of Virology, National Institute of Biomedical Research (INRB), Kinshasa, Democratic Republic of Congo; 9 Department of Clinical Microbiology, Aga Khan University Hospital, Nairobi, Kenya; 10 Department of Medicine, Gastroenterology and Hepatology Section, Baylor College of Medicine, Houston, Texas, USA; 11 Research Center for Global and Local Infectious Diseases, Oita University, Yufu, Japan; 12 Division of Gastroentero-Hepatology, Department of Internal Medicine, Faculty of Medicine-Dr. Soetomo Teaching Hospital, Universitas Airlangga, Surabaya, Indonesia; University of Nebraska Medical Center College of Medicine, Omaha, Nebraska, USA

**Keywords:** amoxicillin resistance, *Helicobacter pylori*, penicillin-binding protein 1A, mutations

## Abstract

The growing resistance to amoxicillin (AMX)—one of the main antibiotics used in *Helicobacter pylori* eradication therapy—is an increasing health concern. Several mutations of penicillin-binding protein 1A (PBP1A) are suspected of causing AMX resistance; however, only a limited set of these mutations have been experimentally explored. This study aimed to investigate four PBP1A mutations (i.e., T558S, N562H, T593A, and G595S) carried by strain KIN76, a high-level AMX-resistant clinical *H. pylori* isolate with an AMX minimal inhibition concentration (MIC) of 2 µg/mL. We transformed a recipient strain 26695 with the DNA containing one to four mutation allele combinations of the *pbp1* gene from strain KIN76. Transformants were subjected to genomic exploration and antimicrobial susceptibility testing. The resistance was transformable, and the presence of two to four PBP1A mutations (T558S and N562H, or T593A and G595S), rather than separate single mutations, was necessary to synergistically increase the AMX MIC up to 16-fold compared with the wild-type (WT) strain 26695. An AMX binding assay of PBP1A was performed using these strains, and binding was visualized by chasing Bocillin, a fluorescent penicillin analog. This revealed that all four-mutation allele-transformed strains exhibited decreased affinity to AMX on PBP1A than the WT. Protein structure modeling indicated that functional modifications occur as a result of these amino acid substitutions. This study highlights a new synergistic AMX resistance mechanism and establishes new markers of AMX resistance in *H. pylori*.

**IMPORTANCE:**

The development of resistance to antibiotics, including amoxicillin, is hampering the eradication of *Helicobacter pylori* infection. The identification of mechanisms driving this resistance is crucial for the development of new therapeutic strategies. We have demonstrated *in vitro* the synergistic role of novel mutations in the *pbp1* gene of *H. pylori* that is suspected to drive amoxicillin resistance. Also deepening our understanding of amoxicillin resistance mechanisms, this study establishes new molecular markers of amoxicillin resistance that may be useful in molecular-based antibiotic susceptibility testing approaches for clinical practice or epidemiologic investigations.

## INTRODUCTION

*Helicobacter pylori* is a Gram-negative bacterium that infects the stomach of approximately 50% of humans (1). Unless treated, this infection persists throughout life and inevitably leads to chronic active gastritis, which is associated with severe gastrointestinal complications including peptic ulcer disease and gastric cancer (2, 3). Despite the understanding that treatment of this infection can heal or prevent such diseases, curing *H. pylori* has proven difficult in some cases because of the limited efficacy of drugs under the harsh physiological conditions of the stomach and the exceptional adaptive abilities of this bacterium (4, 5). *H. pylori* has developed growing resistance to available antibiotics, causing its listing by the World Health Organization as one of the top 20 priority infections that pose the greatest threat to human health due to their drug resistance (6). Therefore, understanding the mechanistic and biological attributes that drive this antimicrobial resistance is crucial to the development of new strategies for overcoming bacterial resistance (7, 8). However, antimicrobial resistance-associated genetic determinants of *H. pylori* have mainly been detected by statistically comparing putative mutations in antibiotic-susceptible and antibiotic-resistant clinical isolates (7, 9). Additional experimental evidence is, therefore, required to establish the biological relevance of resistance attributes for *H. pylori*.

Amoxicillin (AMX) is a major β-lactam antibiotic that is used in *H. pylori* eradication therapy*,* with widely acknowledged efficacy (10). Unlike most bacterial species, AMX resistance (AMX-R) in *H. pylori* has long been considered a rare phenomenon, which explains the limited efforts in exploring its underlying mechanisms (11). Mutations, in particular those altering genes encoding penicillin-binding proteins (PBPs), have been suspected of being the cause of AMX-R, but evidence of their biological relevance is still lacking (7, 9). A recent increase in the epidemiological and clinical significance of this resistance has refocused attention on the need for additional research in this area. Substantial AMX-R has been reported in several regions of the world (12, 13), and clinical strains displaying a minimal inhibitory concentration (MIC) of AMX above 1 µg/mL (which was historically rare) are increasing in prevalence (14, 15). We previously reported a clinical *H. pylori* strain from the Democratic Republic of the Congo exhibiting high-level AMX-R. Following whole genome sequencing (WGS), we identified a set of novel PBP1A amino acid substitutions, including T558S and N562H, in addition to the previously reported but not experimentally established T593A and G595S substitutions (16). These substitutions were suspected of contributing to the AMX-R. In the current study, we aimed to experimentally explore the biological relevance of these mutations in a standard laboratory *H. pylori* strain 26695 by site-specific mutagenesis (SSM), followed by an AMX binding assay and prediction of the PBP1A 3D structure.

## RESULTS

### Synergistic effects of cumulated substitutions in PBP1A mediate amoxicillin resistance

The *H. pylori* strain KIN76 isolated from a patient in the Democratic Republic of the Congo with unusually high resistance to AMX presented four mutations associated with AMX resistance within the *pbp1* gene (i.e., T593A, G595S, T558S, and N562H) (16). Using Cefinase paper discs, we did not detect any β-lactamase activity for the DNA donor strain KIN76, or strain 26695, which was used as the recipient in the following transformation experiments. The MICs of AMX for strains KIN76 and 26695 were 2 and 0.0625 µg/mL, respectively. The *pbp1* gene of strain KIN76 has 87 nucleotide mismatches compared to the *pbp1* of strain 26695. Most of these are synonymous variations, except for 19 amino acid variations that could potentially be associated with AMX-R, including the four alleles mentioned above.

**Fig 1:**
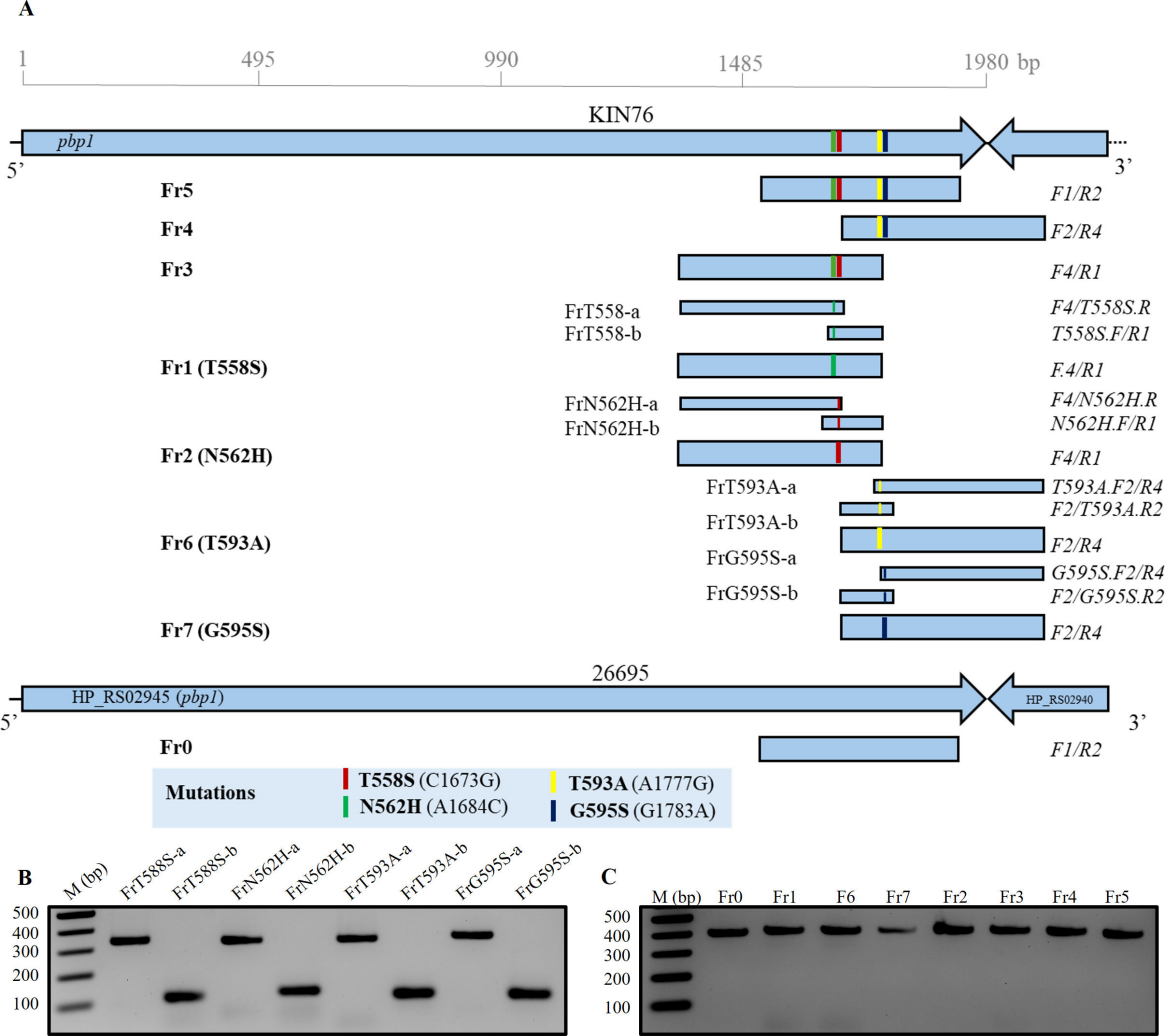
Amplicon construction. The *pbp1* DNA fragments carrying different rearrangements of mutations were amplified by PCR from genomic DNA of clinical isolate KIN76 (**A**). Amplicons Fr1, Fr2, Fr6, and Fr7 carrying a single mutation were additionally constructed by fusion PCR of the single-stranded DNA fragments FrT558S-a/FrT558S-b, Fr2a/Frb2, FrT593A-a/FrT593A-b, and G595S-a/G595S-b, respectively. A DNA fragment (Fr0) without any mutation was also prepared from 26695 genomic DNA and used as a negative control. The PCR fragments before fusion PCR (**B**). All the final KIN76 amplicons prepared before transformation into strain 26695 (**C**). All the PCR fragments were checked in 1.8% agarose S gel electrophoresis.

We used the corresponding ~400-bp DNA fragment in the *pbp1* gene, amplified from the genomic DNA of strain KIN76 directly by polymerase chain reaction (PCR) or constructed in two steps by fusion PCR, to perform the SSM in the recipient AMX-susceptible (AMX^S^) strain 26695 (Fig. 1; Table 1). These DNA fragments carried single (Fr1 with T558S, Fr2 with N562H, Fr6 with T593A, and Fr7 with G595S), dual (Fr3 with T558S/N562H, Fr4 with T593A/G595S), or quadruple (Fr5 with T558S/N562H/T593A/G595S) mutations. Additionally, a PCR product (Fr0) with no mutations obtained from the AMX^S^ strain was used as a control. After the transformation of strain 26695 using Fr0, Fr1, Fr2, Fr6, or Fr7 PCR products, no AMX-resistant (AMX^R^) colonies were detected on AMX plates with a concentration of 0.25 µg/mL or more. By contrast, transformation with dual-mutation carrying fragments Fr3 and Fr4 resulted in AMX^R^ 26695_Fr3 and 26695_Fr4 colonies on 0.25 but not 0.5 µg/mL AMX-containing plates, with an average transformation efficiency of 2.7 × 10^3^ and 3.5 × 10^3^ transformants/µg DNA in three independent experiments, respectively. The AMX MIC of colonies selected from AMX-containing plates ranged from 0.25 to 0.5 µg/mL (Table 2). Similarly, AMX^R^ 26695_Fr5 quadruple-mutation transformant strains were selected on both 0.25 and 0.5 µg/mL AMX-containing plates, with a transformation efficiency of 5.4 × 10^3^ and 3.6 × 10^3^ transformants/µg, respectively (Table 2). The AMX MIC of colonies 26695_Fr5 (with four mutations on PBP1A) ranged from 0.25 to 1 µg/mL, showing higher AMX MICs than colonies 26695_Fr3 and 26695_Fr4 (with two mutations) (Table 3). The resistance phenotype remained consistent after storing transformant and wild-type (WT) strains at −80°C for 2 weeks and after passing through culture on agar plates without AMX.

**TABLE 1:** The sequences and binding sites of primers used in this study^*a*^

Primer name	Sequence (5′–3′)	Binding site
Forward		
F1	CGAAGTCAAAACTTTCACGCCCATTGAAAC	1,521…1,550
F2	TTGACGCTTGGTTCATTGGCTTTACC	1,688…1,713
F3	ATTACGGCACCATGCTCAAACCC	1,466…1,488
F4	CTTTAAGCGACATGGGGTTTAAAAACCT	1,358…1,385
N562H.F	ATTGCCGGTAAAACCGGGACTTCTAACAACCATATTGACG	1,654…1,693
T558S.F	AACCGGGAGTTCTAACAACAATATTGACGCTTGGTTCATT	1,665…1,704
T593A.F2	GGAGCGGCAGGAGGCGTTGTGAGCGCGCCTGT	1,771…1,802
G595S.F2	ACCTATTGGCAAAGGAGCGACAGGAAGCGTTGTGAGCGC	1,758…1,796
Reverse		
R1	CGCTCCTTTGCCAATAGGTGTGT	1,754…1,776
R2	GATGGAATTGGGAGTTGAATAGTAGGGGAT	1,900…1,929
R4	GCGAAGGGTTGCATAAAATGTCTTTAGACG	2,075…2,104
N562H.R	CGTCAATATGGTTGTTAGAAGTCCCGGTTTTACCGGCAAT	1,654…1,693
T558S.R	AATGAACCAAGCGTCAATATTGTTGTTAGAACTCCCGGTT	1,665…1,704
T593A.R2	CGCTCACAACGCCTCCTGCCGCTCCTTTGCCAATAGGT	1,758…1,795
G595S.R2	GCGCTCACAACGCTTCCTGTCGCTCCTTTGCCAATAGGT	1,758…1,795

^*a*^ The nucleotides underlined indicate the substitutions incorporated into the primer sequences. These modifications were specifically devised to swap the mutated nucleotide found in the *pbp1* gene of the resistant strain KIN76 with the corresponding nucleotide from the susceptible strain 26695, in order to produce fused amplicons harboring each a single mutation.

**TABLE 2:** Transformation efficiency of strain 26695 with PCR products of the *pbp1* gene from AMX^R^ strain KIN76

Fragment	Mutated allele(s)	AMX concentration on plate (µg/mL)	Transformation efficiency^*a*^
No DNA control	None	0.125	>10^4^
		0.25	0
Fr0	None	0.125	>10^4^
		0.25	0
Fr1	T558S	0.125	>10^4^
		0.25	0
Fr2	N562H	0.125	>10^4^
		0.25	0
Fr6	T593A	0.125	>10^4^
		0.25	0
Fr7	G595S	0.125	>10^4^
		0.25	0
Fr3	T558S/N562H	0.25	2.7 × 10^3^
		0.5	0
Fr4	T593A/G595S	0.25	3.5 × 10^3^
		0.5	0
Fr5	T558S/N562H/T593A/G595S	0.125	>10^4^
		0.25	5.4 × 10^3^
		0.5	3.6 × 10^3^

^*a*^ Transformation efficiency (transformants/microgram DNA) calculated based on the formula shown in Materials and Methods.

**TABLE 3:** AMX MICs of the wild-type strains (at baseline) and the transformants

Strain type	Mutated alleles	Strain	AMX MIC (µg/mL)
WT DNA recipient		26695	0.0625
WT DNA donor		KIN76	2
		Transformant by amplicon	
Fr3	T558S/N562H	26695_Fr3-1	0.25
		26695_Fr3-2	0.25
		26695_Fr3-3	0.25
		26695_Fr3-4	0.25
		26695_Fr3-5	0.25
		26695_Fr3-6	0.5
		26695_Fr3-7	0.5
		26695_Fr3-8	0.25
Fr4	T593A/G595S	26695_Fr4-1	0.5
		26695_Fr4-2	0.5
		26695_Fr4-3	0.25
		26695_Fr4-4	0.5
		26695_Fr4-5	0.25
		26695_Fr4-6	0.5
		26695_Fr4-7	0.25
		26695_Fr4-8	0.25
Fr5	T558S/N562H/T593A/G595S	26695_Fr5-1	0.5
		26695_Fr5-2	1
		26695_Fr5-3	0.5
		26695_Fr5-4	0.25
		26695_Fr5-5	1
		26695_Fr5-6	1
		26695_Fr5-7	0.5
		26695_Fr5-8	1

### Mutated alleles of the *pbp1* gene in transformants

**Fig 2:**
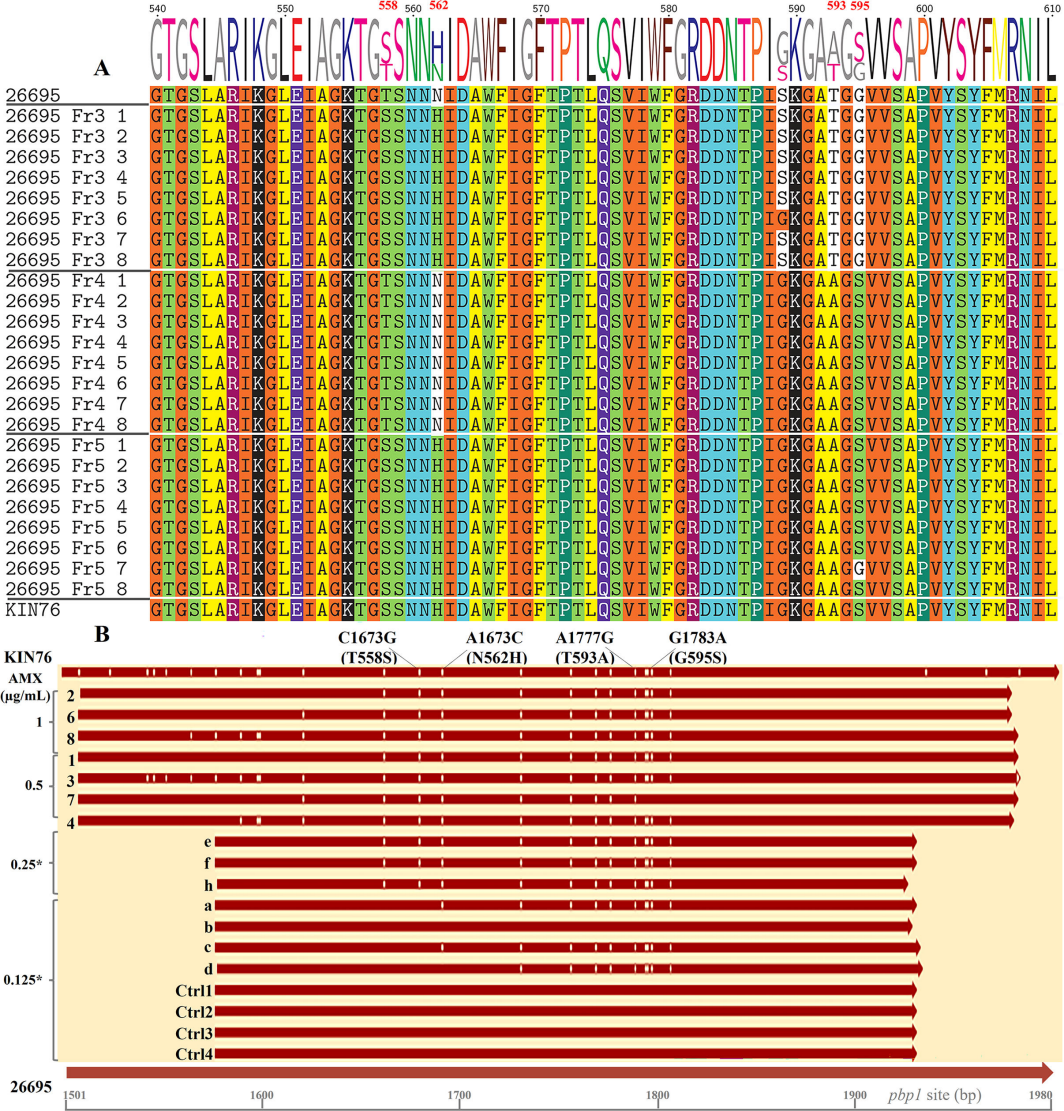
Alignment of PBP1A amino acid and *pbp1* nucleotide sequences. (**A**) Alignment of PBP1A amino acid sequences. After Sanger sequencing and initial processing of the raw data, the *pbp1* gene fragments from transformants were aligned with sequences from the WT 26695 strain and the KIN76 clinical strain, then translated into protein sequences. T558 and N562 of strain 26695 were substituted by S and H in all transformant strains obtained by transformation with the Fr3 DNA fragment, except one strain (26695_Fr3-6) that contained not-AMX-R-related G589, same as strain KIN76. T593 and G595 of strain 26695 were substituted by A and S in transformant strains obtained by transformation with the Fr4 DNA fragment in all strains. Similarly, in transformants resulting from transformation with the Fr5 DNA fragment, substitutions occurred on all four loci in every strain sequenced except for one strain (26695_Fr5-7) that showed a conserved G595. (**B**) Nucleotide sequences of Fr5 and control strains. All the Fr5 strains (Fr5-1 to Fr5-8) were transformed using the Fr5 amplicon, and their MICs, determined later, ranged from 0.25 to 1 µg/mL. The asterisk indicates that each colony of the strain was selected on a plate containing AMX at the indicated concentration. Sequences Fr5-05 (e, f, and h) derive from strains selected on plates containing 0.5 µg/mL AMX, whereas sequences Fr5-0125 (a–d) are from Fr5-transformant strains picked from non-selective plates with 0.125 µg/mL AMX. Control sequences (Ctrl1 to Ctrl4) are from control colonies picked from non-selective plates. All sequences were aligned to the *pbp1* gene of WT strains 26695 and KIN76, spanning nucleotides 1,501 to 1,980. Vertical lines within the sequences denote nucleotide variation sites in comparison to the 26695 sequence. Mutation sites are indicated at the top of the figure.

In each experiment, the existence of mutations in the *pbp1* gene was confirmed in eight randomly selected colonies of AMX^R^ transformants by Sanger sequencing of the *pbp1* gene. All eight 26695_Fr3 strains contained front dual mutations, all eight 26695_Fr4 strains contained rear dual mutations, and seven of the eight 26695_Fr5 strains contained quadruple mutations, which was consistent with the original PCR fragments before SSM (Fig. 2A). Interestingly, one of the 26695_F5 strains, strain Fr5-7, displayed only three of the four expected amino acid substitutions, lacking the last mutated allele S595 and possessing the original G595 instead. We aligned the original *pbp1* sequences with the corresponding regions of Fr5 AMX^R^ transformants, as well as with sequences from strains transformed using the Fr5 amplicon and selected on plates containing 0.5 µg/mL AMX (Fig. 2B). Additionally, we included sequences from strains transformed with the same amplicon but picked from non-selective plates with 0.125 µg/mL AMX, and control strains picked from non-selective plates. None of the aligned control *pbp1* sequences exhibited nucleotide variation compared to the 26695 *pbp1*. However, the Fr5 sequences showed some nucleotide variations, likely due to differences in the corresponding homologous recombination sites. All AMX^R^ strains (Fr5-1 to Fr5-8) exhibited all four mutations, except for 26695_Fr5-7 mentioned above. Fr5 colonies picked from non-selective plates showed either two (Fr5-0125d) or three (Fr5-0125a and Fr5-0125c) mutations, while Fr5-0125b exhibited no mutation. Despite observing some variations beyond the four mutations under study, all were consistent with the nucleotides in the WT *pbp1* of the DNA donor strain KIN76. This indicates that *H. pylori*, due to its natural competence, can incorporate environmental DNA, with possible genomic rearrangements following the DNA uptake. However, the bacterium still needs to accumulate specific critical mutations to survive under high antibiotic concentration conditions. The analysis of these sequences also indicates that the other variations, particularly those upstream of the 1,650 bp site and downstream of the 1,850 bp site, do not contribute to a higher increase in AMX MIC (beyond 0.5 µg/mL, for example). Since the 26695_Fr5-7 strain showed an MIC of 0.5 µg/mL, consistent with the MIC of some Fr5 strains carrying four mutations (Table 3), the simultaneous presence of at least the first three mutations would be sufficient to induce AMX resistance as high as 0.5 µg/mL.

At position 589 of PBP1A, strain 26695_Fr3-6 exhibited glycine (G), while all other seven screened strains in this Fr3 set showed serine (S) (Fig. 2A), indicating that induced mutagenesis may result in spontaneously acquired genetic mutations at a low frequency. To explore the occurrence of such mutations in other loci, we generated and analyzed the WGS data of transformant 26695_Fr3-8, carrying induced T558S and N562H mutations in PBP1A. This analysis focused on genes encoding PBP1A, PBP2, and PBP3, as well as additional components of the bacterial divisome or elongasome (i.e., MreC, LpoB, MurJ, FtsX, FtsZ, and FtsW) interacting with PBPs, and which could interfere with the antimicrobial activity of AMX (17–20). Except for the induced T558S and N562H mutations in PBP1A, no spontaneous mutations were found in different genomic loci of 26695_Fr3-8 compared to sequences retrieved from WGS of AMX^S^ strains 26695 and J99 (21) (Tables S8 to S17). Overall, this analysis suggested, on the one hand, that T558S and N562H mutations would induce AMX-R, in addition to the previously reported T593A and G595S mutations (22); on the other hand, it also suggested a minimal impact of possible mutations spontaneously acquired in AMX-R-related genes on reported outcomes. However, further investigations are needed to deepen our understanding of the biological significance of spontaneous mutations following mutagenesis in *H. pylori*.

From the genetic evaluation, overall, the accumulation of T558S, N562H, T593A, and G595S amino acid substitutions in the *pbp1* gene was responsible for the high-level AMX-R of strain KIN76. Furthermore, the presence of at least two PBP1A mutations (T558S and N562H, or T593A and G595S) rather than separate single mutations was shown to be necessary to synergistically induce a mild AMX-R. The AMX-R conferred by these mutations was demonstrated to be horizontally transferable to susceptible strains.

**Fig 3:**
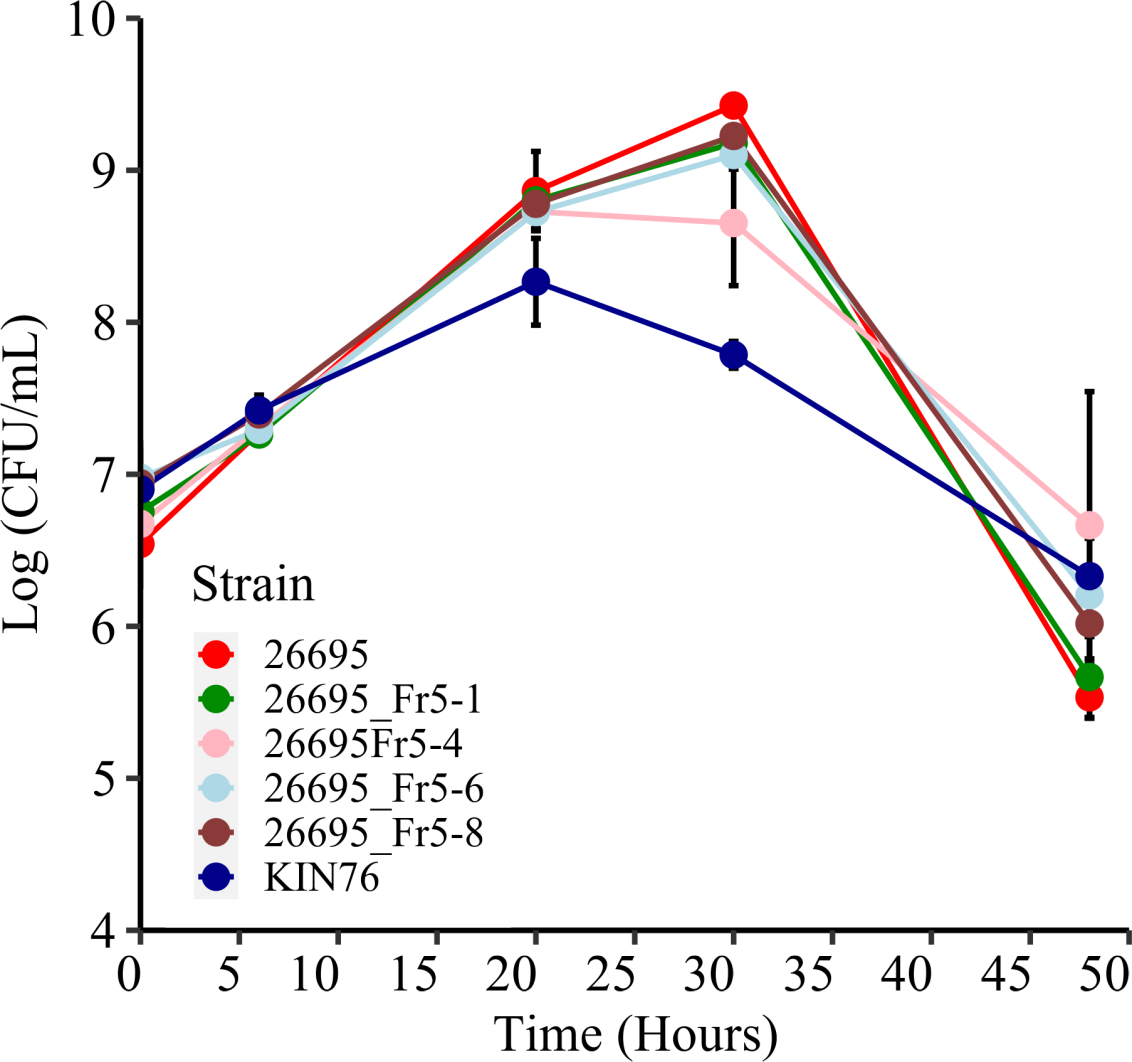
Growth rate of WT and Fr5 mutant strains. This figure depicts the growth rates of the WT strain KIN76 and the AMX-R mutant strains 26695_Fr5-1, 26695_Fr5-4, 26695_Fr5-6, and 26695_Fr5-8 compared to the susceptible strain 26695. Colony-forming units (CFUs) were counted at 0, 6, 20, 30, 48, and 72 hours post-inoculation. The results are presented as the means and standard errors of four independent experiments. The mutant strains did not exhibit a significant difference from the WT 26695, whereas the growth rate of strain KIN76 showed a significant difference compared to 26695 by the Wilcoxon signed-rank test (*P* = 0.0264).

However, none of the transformed strains that newly acquired AMX-R exhibited a resistance level equivalent to that of the WT strain, the DNA donor KIN76. Moreover, within a set of strains harboring identical mutations in *pbp1*, some variations in MICs were noted, suggesting the probable involvement of additional resistance mechanisms. The potential impact of the bacterial growth rate on antibiotic susceptibility has been discussed in previous studies (23, 24). Additionally, we have observed a comparatively sluggish growth of the strain KIN76 in comparison to 26695. Therefore, we selected four transformed strains harboring all four mutations and showing AMX MICs ranging from 0.25 to 1 µg/mL (26695_Fr5-1, 26695_Fr5-4, 26695_Fr5-6, and 26695_Fr5-8), alongside the WT strains KIN76 and 26695, for liquid culture growth analysis. The growth rates were evaluated and compared to the susceptible strain 26695 (Fig. 3). While strain KIN76 showed a notably different growth pattern compared to strain 26695 (*P* = 0.0264), the mutated Fr5 strains derived from 26695 displayed individual growth variations that were not statistically significant when compared to the WT strain. Thus, the observed AMX-R in the four allele-substituted strains did not appear to be correlated with the growth rate.

### Decreased AMX binding affinity of PBP1A with dual and quadruple mutations

**Fig 4:**
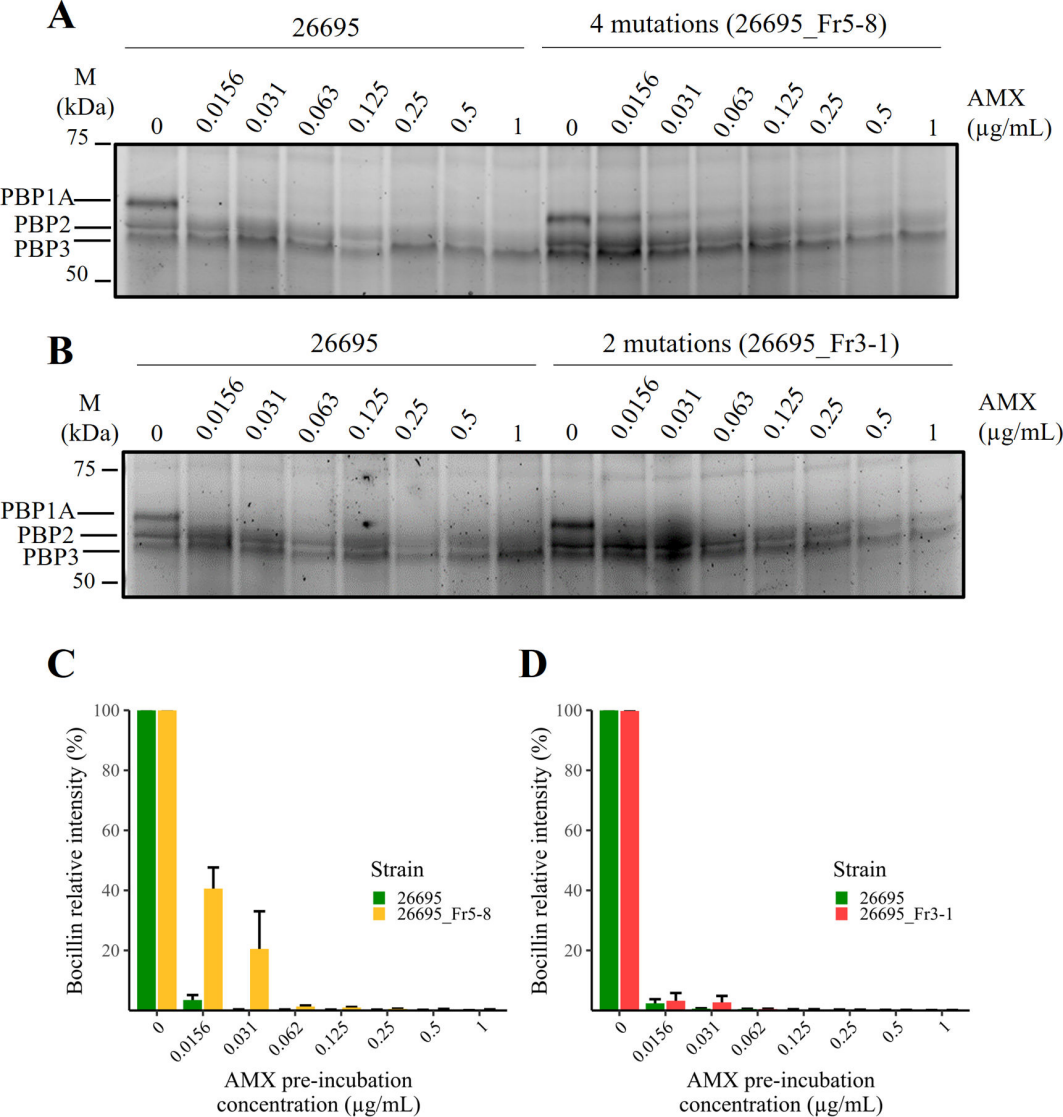
AMX-Bocillin competitive binding assay. The relative signal intensity of Bocillin was set to maximal when no AMX was added to the binding assay reaction (AMX preincubation concentration = 0). However, preincubation with 0.0156–0.031 µg/mL of AMX showed a higher Bocillin signal in transformant 26695_Fr5-8 (**A, C**) than transformant 26695_Fr3-8 (**B, D**), indicating a decrease in PBP1A affinity for AMX in transformants carrying mutations within PBP1A, with a greater effect seen with PBP1A harboring four mutations than PBP1A harboring only two mutations. The total protein intensity for each lane (not shown) was used for Bocillin quantification. Three independent assays were performed (*n* = 3).

To explore the impact of the four PBP1A mutations in the AMX-R, we performed an AMX binding competitive assay using Bocillin, which is a fluorescent compound and an analog of penicillin V used as a readout probe to study the profile of PBPs in bacteria (25). Using *H. pylori* strain 26695, we first confirmed how many PBPs can be detected by Bocillin. For this purpose, we used a recently reported method based on live-cell profiling of PBPs using a buffer containing ethylenediamine-tetra acetic acid (EDTA) to permeabilize the outer membrane of Gram-negative bacterial cells via lipopolysaccharide disruption (26). *H. pylori* bacterial cells were preincubated in AMX at a final concentration of 0.01–67 µg/mL in Tris-EDTA buffer. The cells were then incubated in 25 µM of Bocillin. SDS-PAGE imaging of the samples revealed three main bands in the range of 50–75 kDa in the control lane (Fig. S1), likely corresponding to PBP1, PBP2, and PBP3, as these bands are consistent with a previous report using biotin-labeled ampicillin (27). When bacterial cells were preincubated in 0.01, 1, and 67 µg/mL AMX before Bocillin addition, the PBP1 band disappeared at 0.01 µg/mL AMX or more, indicating that AMX at this concentration was fully covalently bound to the binding sites of PBP1A, excluding the binding of Bocillin. Therefore, PBP1 shows a higher affinity to AMX than PBP2 and PBP3 whose bands remained observable even at 67 µg/mL of AMX, indicating a low affinity for AMX that could bind PBP1. The PBP1 band was identified as the PBP1A protein, encoded by the *pbp1* gene, based on its predicted molecular weight of 74.3 kDa (Table S1). Consequently, we performed the AMX competitive assay, to evaluate the binding affinity of mutated PBP1A proteins to AMX. The affinity of AMX for PBP1A was compared between each of the transformants, 26696_Fr5-8 (carrying T558S, N562H, T593A, and G595S mutations, with an AMX MIC of 1 µg/mL) or 26695_Fr3 (carrying T558S and N562H mutations, with an AMX MIC of 0.5 µg/mL), and the WT AMX^S^ strain *H. pylori* 26695. In the four-mutation transformant strain 26695_Fr5-8, at AMX preincubation concentrations of 0.0156 and 0.031 µg/mL, stronger Bocillin signals on PBP1A were detected than in the WT 26695. This suggests either a reduced binding affinity of Bocillin by the PBP1A of 26695_Fr5-8 in comparison to the WT strain or an equivalent level of binding with weaker interaction strength (Fig. 4A). T558S and N562H transformant strain 26695_Fr3-1 displayed slightly elevated Bocillin signals on PBP1A at 0.0156 µg/mL AMX but not at higher AMX concentrations (Fig. 4B). The quantified results of PBP1A bands (Fig. 4C and D) clearly indicated that AMX binding affinity tends to decrease as the number of PBP1A mutations increases from two to four. These results indicated that the decreased AMX binding affinity of PBP1A of the strains was caused by transformed DNA fragments including four mutations in the *pbp1* gene (T558S, N562H, T593A, and G595S) transferred from KIN76 into the strain 26695, and this is one of the mechanistic reasons for the AMX-R phenotype observed in KIN76.

### Prediction of the 3D structure of PBP1A

**Fig 5:**
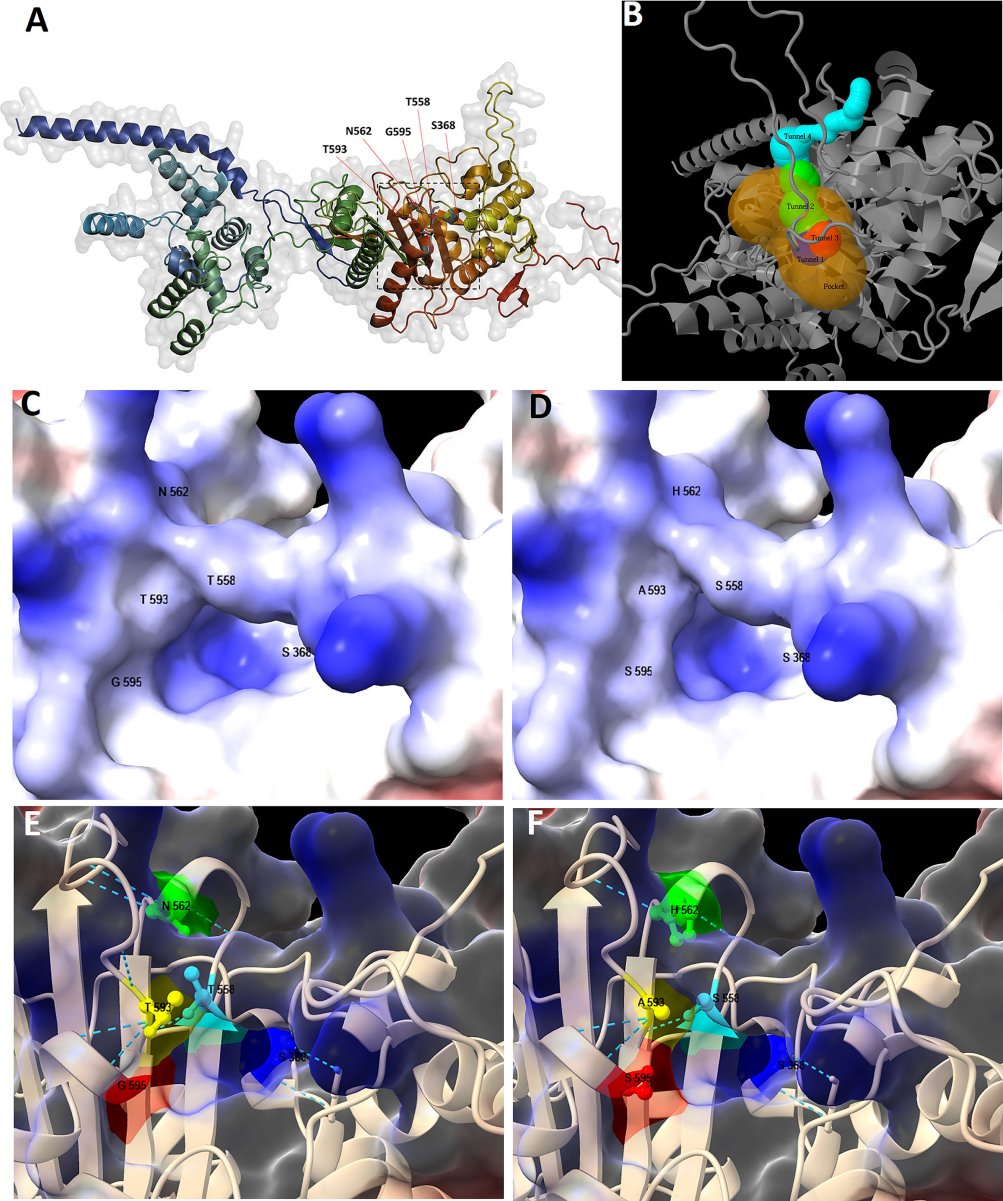
*H. pylori* PBP1A tertiary structure models. Whole structure of PBP1A of strain 26695, modeled through Alfafold2Colab and visualized using PyMol (**A**); the square roughly indicates the pocket, and the red arrows indicate residues at positions 368, 558, 562, 593, and 595. The pocket with the highest relevance score, centered around catalytic residue S368 (hidden in the figure), and the corresponding tunnels as predicted by Caver (**B**). Closed-up structures of PBP1A near the catalytic residue were visualized without mutation (**C, E**) and with four mutations (**D, F**) using CSF-Chimera. In surface models (**C, D**), regions of positive charge are shaded blue (basic), and those of negative charge are shaded red (acidic). In ribbon models (**E, F**), residues in positions 558 (light green), 562 (orange yellow), 593 (orange), and 595 (red) are shown to line to the access route to catalytic residue S368 (blue). The narrow green line traces the approximate contour of the residues, and dotted lines correspond to hydrogen bonds.

To understand the reduced binding affinity in the transformants, we predicted the tertiary structure of PBP1A in strain 26695 and the transformant harboring four mutations. First, a 3D model of PBP1A of strain 26695 without mutations was built using Alfafold2Colab and visualized with PyMol (Fig. 5A). The serine at position 368 was identified as the catalytic residue by both Caver (Table S2) and Cofactor tools, which was in line with previous studies that reported S368 as penicillin-binding and transpeptidase catalytic sites (28, 29). The Caver tool found four tunnels and a pocket, centered around the catalytic residue (Fig. 5B; Tables S3 and S4). The Cofactor tool also predicted nine residues, including that at position 558, as being part of the antibiotic binding site (Table S4). Interestingly, the residues at positions 558 and 562, as well as those at positions 593 and 595, were found to be shared by all of the predicted tunnels (Table S5).

The comparison of the 3D model of PBP1A without mutations to that containing four mutations revealed a slight modification in the surface shape around the catalytic residue S368 (Fig. 5C and D). In the mutated protein, the area surrounding the catalytic residue S368 exhibited reduced basicity, particularly in the vicinity of the asparagine (N) to histidine (H) substitution at position 562. This change resulted in a bulging effect due to the replacement of asparagine with the larger, positively charged and imidazole-containing histidine. Additionally, the substitution of the small glycine (G) with serine (S) at position 595 further contributed to this structural alteration (Fig. 5C vs Fig. 5D). Moreover, the three hydrogen bonds formed by asparagine (N562) were modified to two bonds in different orientations due to its substitution by histidine (H562) (Fig. 5E vs Fig. 5F). Considering these observations, we propose that these amino acid changes alter the access of AMX to the binding site and/or the AMX binding affinity itself, subsequently leading to a decrease in PBP1A binding affinity by modifying the entry tunnel and/or structure around the AMX binding site.

## DISCUSSION

In the current study, we explored the biological relevance of mutations detected in *pbp1* gene of KIN76, a high-level AMX^R^ *H. pylori* strain isolated from a Congolese patient. The SSM was performed, followed by an AMX affinity binding assay and 3D protein structure analysis.

We transformed the recipient *H. pylori* 26695 using amplicons from the DNA donor strain KIN76. The highly efficient system mirroring homologous natural transformation, which is well established for *H. pylori* (30–32), yielded transformant strains with a higher AMX MIC than the MIC of the recipient strain. We accumulated evidence that AMX-R has mainly been associated with point mutations in the PBP1A, a class A bifunctional PBP having transglucosylase and transpeptidase activities and involved in peptidoglycan metabolism, although the effects of only a few of these mutations have been experimentally established (27, 33, 34). Like most β-lactam antibiotics, AMX mimics the D-alanyl-D-alanine dipeptide and acts as a suicide inhibitor by covalently binding the transpeptidase catalytic site. During the binding of AMX to PBP, the β-lactam ring is attacked by the catalytic residue S368 and opened, which results in the formation of a covalent acyl-enzyme complex that is slowly hydrolyzed (29). Covalently bound AMX prevents the transpeptidase activity of PBP required for the polymerization of glycan chains, which deprives the bacterial cell of a process essential for the integrity of its cell wall and its viability (7). The mutations analyzed in this study would, therefore, impart modifications in PBP1A that have inhibitory effects on AMX binding.

Interestingly, the DNA donor isolate KIN76 exhibits a MIC of 2 µg/mL, whereas strains bearing all four PBP1A mutations following transformation display MICs ranging from 0.25 to 1.0 µg/mL. The inability of these strains to reach the MIC of KIN76 suggests the influence of additional genetic and phenotypic factors. Firstly, beyond the four mutations studied, the KIN76 PBP1A accumulated 15 amino acid variations that could have further increased AMX-R in the donor strain. Secondly, mutations in non-*pbp1* loci might have contributed to the increased AMX-R, including those in proteins such as PBP2, PBP3, FtsW, FtsX, FtsZ, LpoB, MreC, or MurJ. These proteins are likely involved in peptidoglycan synthesis in *H. pylori* through their transpeptidase and glycosyltransferase activity or their activities as major components of cell divisome and elongasome, as demonstrated in several bacterial species (17–20). Genetic mutations or dysregulation in the expression of these proteins could further impact the antimicrobial activity of AMX and interact with PBP1A mutations, contributing to higher AMX-R in KIN76. Further functional investigations are needed to provide insights into these proposed mechanisms of AMX-R in the *H. pylori* species.

Furthermore, a significant phenotypic difference between 26695 and KIN76 is their growth rates, which could potentially impact AMX-R. β-Lactam antibiotics are most effective during the exponential growth phase of bacteria, as they inhibit cell membrane synthesis during cell division (23, 24, 35). Hence, their activity might be reduced in bacteria that grow slowly. KIN76 turned out to be a slow-growing strain, with a low-amplitude exponential phase, which may contribute additionally to its higher-level resistance and partially account for the differences in MIC levels. However, it is worth noting that clinical strains of *H. pylori* tend to grow more slowly than laboratory strains. Moreover, *H. pylori* species are known for their high genetic variability, which may further explain the observed differences between mutants derived from the same parental strain, notwithstanding harboring identical mutations in *pbp1* gene (36).

Following induced mutagenesis, three out of the four anticipated substitutions were identified in a single strain after selection on plates with a high AMX concentration, indicating genetic recombination subsequent to the integration of the transferred DNA (30). Further WGS of a transformant that successfully acquired T558S and N562H in PBP1A revealed no additional genetic mutations in the *pbp1* gene or the other two *pbp* genes that have been linked to AMX-R. Moreover, no mutations were found in genes encoding the FtsW, FtsX, FtsZ, LpoB, mreC, and MurJ proteins that likely interact with PBPs in the divisome or the elongasome during bacterial cell wall construction and maintenance (7, 9, 17–20). This underscores the significance of the newly acquired mutations to the development of AMX-R.

Inducing single T558S, N562H, T593A, or G595S mutations in the *pbp1* gene did not result in AMX-R in this study. However, the *pbp1* mutation N562Y has been associated with AMX-R in clinical isolates from Mongolia (37) and Japan (38) and in experimental studies (39). Unlike single mutations, when both T558S and N562H or T593A and G595S substitutions were combined, AMX MICs reached moderately higher levels than those in the recipient strain. Introducing further mutations (i.e., T558S, N562H, T593A, and G595S in combination) resulted in even higher MICs compared with the original WT strain 26695. The synergistic aspect of this resistance suggests that, in the presence of subclinical levels of AMX, the bacterium could incrementally acquire non-critical mutations individually, which could confer resulting high-level resistance. Such synergistic effects of accumulated mutations on the phenotype, particularly antibiotic resistance, have also been observed in other bacterial species as well (40, 41). Moreover, the acquired resistance in this study was found to be stable, in contrast to the putatively unstable AMX-R of *H. pylori*, which has been previously reported to be associated with the reduced expression of PBP-D (42).

To further uncover the molecular effects of the studied mutations, we showed that they decreased the binding affinity of PBPs for AMX using the Bocillin probe. This involved a series of AMX incubations to visualize the non-bound AMX in the WT and mutant PBP1A. Only PBP1A exhibited reduced signals depending on the AMX preincubation concentration. In the WT strain 26695, a reduction in the Bocillin signal was achieved with lower amounts of AMX whereas a higher AMX concentration was required in transformants with two or more PBP1A mutations. The loss of binding of AMX in transformants suggests that mutations are conferring two possible inhibitory activities from the 3D prediction: (i) modification of AMX binding itself to the catalytic residue S368 by the structural modification of the surrounding area or (ii) structural alteration of the AMX access route, or possibly both. These activities are plausible since the amino acid substitutions are located adjacent to two of the three known penicillin-binding motifs of PBP1A (i.e., SAIK_368–371_, KTG_555–557_, and SNN_559–561_) (7) and line the access route to the hotspot catalytic site of the enzyme (i.e., S368). In addition, the T558 residue is typically predicted to be involved in hydrogen bond interactions with the catalytic site and in covalent complex interactions for AMX binding to S368 (29). Furthermore, the amino acid substitutions resulted in protein surface and chemical property modifications.

Taken together, these amino acid changes synergistically result in protein conformation change, which would alter the access and binding of AMX to PBP1A. However, caution should be taken when generalizing these conclusions to all possible mutations of *pbp1*, considering that the codon at each position may have its own functional importance. This further stresses the need to validate several mutations that are suspected to confer resistance to AMX in *H. pylori* but have not yet been functionally investigated (9). In this study, we established new molecular markers of AMX-R that may be a useful contribution to molecular-based antimicrobial susceptibility testing approaches, which are increasingly being applied in clinical and epidemiological practice.

## MATERIALS AND METHODS

### *H. pylori* strains

*H. pylori* strain KIN76 was previously isolated from a patient in the Democratic Republic of the Congo with an unusually high resistance to AMX (16) and belongs to the *hpAfrica1* population. *H. pylori* 26695, initially obtained from the American Type Culture Collection, was originally isolated from a patient in the United Kingdom (43) and belongs to the *hpEurope* population (Fig. S2).

### Bacterial culture and drug susceptibility testing

*H. pylori* strains were stored at −80°C and cultured on Brucella agar plates supplemented with 7% horse blood by incubation under microaerobic conditions. AMX susceptibility was evaluated using the agar dilution method, and the determination of MICs was conducted according to the Clinical and Laboratory Standards Institute (Wayne, PA, USA) protocol (44). Briefly, twofold dilutions of AMX (Wako Pure Chemical Industry, Chuo-ku, Osaka, Japan) were prepared in Müller–Hinton agar supplemented with 5% horse blood. Two-day subcultured bacteria were suspended in Brucella broth supplemented with 10% horse serum and adjusted to an optical density at 590 nm (OD_590_) of 0.1, then spotted onto Müller–Hinton agar plates using a 1 µL pin inoculator. The MICs were determined 72 hours later. The breakpoint for defining AMX-R was set to 0.125 µg/mL. The test was performed in duplicate and repeated. β-Lactamase activity was checked by the BD BBL Cefinase 50 (BD, Sparks, MD, USA).

### DNA extraction and construction of amplicons for natural transformation

*H. pylori* genomic DNA was extracted using the DNeasy Blood and Tissue Kit (Qiagen, Hilden, Germany) according to the manufacturer’s guidelines and quantitated using the Quantus Fluorometer (Promega, Madison, WI, USA). The *pbp1* gene of *H. pylori* strain KIN76 was PCR-amplified using PrimeSTAR GXL Premix (Takara Bio Inc., Otsu, Shiga, Japan) with sets of primers designed using SnapGene software v.6.0 (45) (Table 1). The amplification consisted of 30 cycles in a thermocycler programmed with the following settings: denaturation at 98°C for 10 seconds, annealing at 51°C for 15 seconds, and extension at 68°C for 45 seconds. PCR amplification enabled the construction of amplicons harboring different arrangements of mutations under study [i.e., fragment Fr0 (419 bp) without mutation, Fr1 (419 bp) with T558S, Fr2 (419 bp) with N562H, Fr6 (417 bp) with T593A, Fr7 (417 bp) with G595S, Fr3 (419 bp) with T558S and N562H, Fr4 (417 bp) with T593A and G595S, and Fr5 (409 bp) with T593A, G595S, T558S, and N562H]. For the fusion PCR to construct only one mutation out of closely related dual mutations for T558S, N562H, T593A, and G595S (Fig. 1A), mutations were designed within the primer sequences. For example, the PBP1A.F4/PBP1A-T558S.R primer pair amplified a 347-nucleotide single-stranded amplicon (FrT558S-a), while another single-stranded amplicon (FrT558S-b) of 112 nucleotides was obtained by combining the PBP1A-T558S.F/PBP1A.R1 primers. Subsequently, these fragments were fused in a second PCR using the PBP1A.F4/PBP1A.R1 primers, yielding a double-stranded 419-bp fragment Fr1 containing a single mutation (T558S) (Fig. 1A). Similarly, the fusion of amplicons Fr2a (336 bp) and FrN562H-b (123 bp) using the PBP1A.F4/PBP1A-N562H.R and PBP1A.R1/PBP1A-N562H.F primers, FrT593A-a (334 bp) and FrT593A-b (108 bp), and FrG595S-a (347 bp) and FrG595S-b (108 bp) produced the amplicons Fr2 (with N562H), Fr6 (with T593A), and Fr7 (with G595S) respectively. Amplicon constructs underwent size verification through electrophoresis in a 1.8% agarose S gel, while their sequences were validated using Sanger sequencing (Fig. 1B and C).

### Natural transformation and phenotype assessment

*H. pylori* 26695 was transformed based on a protocol described by Haas et al. (46), with some modifications. Briefly, a 2-day subcultured strain was suspended in Brucella broth supplemented with 10% fetal bovine serum (FBS) with a starting OD_590_ of 0.1. Then, it was dispensed into a six-well plate (1 mL/well) and incubated for 6 hours under microaerobic conditions until the OD_590_ reached 0.3. Then, 0.1 µg of the PCR-amplified DNA fragment was mixed with the liquid culture. Following an 18-hour incubation, bacterial cultures were spread onto selective Brucella agar plates supplemented with 10% FBS and 0.125–0.5 µg/mL AMX (Wako Pure Chemical Industries). An AMX-free plate was included as a control. The transformation efficiency, expressed as the number of transformants per microgram DNA, was calculated based on the following equation and presented as the mean of three independent experiments.


$$Transformation\;efficiency=\frac{Number\;of\;transformants}{Bacterial\;suspension\;volume\;inoculated\;on\;selective\;plates(mL)}\times \frac{Total\;bacterial\;suspension\;volume\;used\;for\;transformation\;(mL)}{DNA\;amount\;(\mu g)}$$

Eight colonies were picked from AMX plates and grown on AMX-free agar plates for further experiments (Fig. S3).

The characteristics of all *H. pylori* strains used in this study are summarized in Table 4.

**TABLE 4:** *H. pylori* strains used in this study

Strain	Description	Reference (accession no.)
Wild types		
KIN76	AMX^R^, carrying mutations T558S, N562H, T593A, and G595S in PBP1A, and used as DNA donor	(16) (OR855674)
26695	AMX^S^, used as DNA recipient	(NC_000915)
Control strains		
Control 1	Picked from a non-selective plate and not carrying mutations	This study
Control 2		This study
Control 3		This study
Control 4		This study
Transformants		
Originating from 26695, transformed with the *pbp1* fragment of KIN76 and selected as AMX^R^		
26695_Fr3-1	AMX^R^, carrying T558S and N562H in PBP1A^26695^	This study
26695_Fr3-2	AMX^R^, carrying T558S and N562H in PBP1A^26695^	This study
26695_Fr3-3	AMX^R^, carrying T558S and N562H in PBP1A^26695^	This study
26695_Fr3-4	AMX^R^, carrying T558S and N562H in PBP1A^26695^	This study
26695_Fr3-5	AMX^R^, carrying T558S and N562H in PBP1A^26695^	This study
26695_Fr3-6	AMX^R^, carrying N562H in PBP1A^26695^	This study
26695_Fr3-7	AMX^R^, carrying T558S and N562H in PBP1A^26695^	This study
26695_Fr3-8	AMX^R^, carrying T558S and N562H in PBP1A^26695^	This study (JAXCGF000000000)
26695_Fr4-1	AMX^R^, carrying T558S and N562H in PBP1A^26695^	This study
26695_Fr4-2	AMX^R^, carrying T558S and N562H in PBP1A^26695^	This study
26695_Fr4-3	AMX^R^, carrying T558S and N562H in PBP1A^26695^	This study
26695_Fr4-4	AMX^R^, carrying T558S and N562H in PBP1A^26695^	This study
26695_Fr4-5	AMX^R^, carrying T558S and N562H in PBP1A^26695^	This study
26695_Fr4-6	AMX^R^, carrying T558S and N562H in PBP1A^26695^	This study
26695_Fr4-7	AMX^R^, carrying T558S and N562H in PBP1A^26695^	This study
26695_Fr4-8	AMX^R^, carrying T558S and N562H in PBP1A^26695^	This study
26695_Fr5-1	AMX^R^, carrying T558S, N562H, T593A, and G595S in PBP1A^26695^	This study
26695_Fr5-2	AMX^R^, carrying T558S, N562H, T593A, and G595S in PBP1A^26695^	This study
26695_Fr5-3	AMX^R^, carrying T558S, N562H, T593A, and G595S in PBP1A^26695^	This study
26695_Fr5-4	AMX^R^, carrying T558S, N562H, T593A, and G595S in PBP1A^26695^	This study
26695_Fr5-5	AMX^R^, carrying T558S, N562H, T593A, and G595S in PBP1A^26695^	This study
26695_Fr5-6	AMX^R^, carrying T558S, N562H, T593A, and G595S in PBP1A^26695^	This study
26695_Fr5-7	AMX^R^, carrying T558S, N562H, and T593A in PBP1A^26695^	This study
26695_Fr5-8	AMX^R^, carrying T558S, N562H, T593A, and G595S in PBP1A^26695^	This study
26695_Fr5-05e	From AMX 0. 5 µg/mL plate, carrying T558S, N562H, T593A, and G595S in PBP1A^26695^	This study
26695_Fr5-05f	From AMX 0. 5 µg/mL plate, carrying T558S, N562H, T593A, and G595S in PBP1A^26695^	This study
26695_Fr5-05g	From AMX 0.5 µg/mL plate, carrying N562H, T593A, and G595S in PBP1A^26695^	This study
26695_Fr5-0125a	From AMX 0.125 µg/mL plate, carrying T558S, N562H, T593A, and G595S in PBP1A^26695^	
26695_Fr5-0125b	From AMX 0.125 µg/mL plate, exhibiting no mutation	
26695_Fr5-0125c	From AMX 0.125 µg/mL plate, carrying T558S, N562H, T593A, and G595S in PBP1A^26695^	
26695_Fr5-0125d	From AMX 0.125 µg/mL plate, carrying T593A, and G595S in PBP1A^26695^	This study

### Growth curves

Frozen *H. pylori* strains were grown on Brucella agar plates supplemented with 7% horse blood. Following a series of plate subculturing steps, 2-day cultured strains were precultured overnight in Brucella broth supplemented with 10% FBS. Subsequently, 20-mL cultures were adjusted to a starting OD_590_ of 0.05 in 200-mL flasks and incubated under 5% CO_2_/37°C conditions with shaking. CFU enumeration and OD measurements were recorded at 0, 6, 20, 30, 48, and 72 hours post-inoculation. The presented results are representative of four biologically independent experiments. The growth curves were plotted using R software (47, 48). Subsequently, the Wilcoxon signed-rank test was applied to compare the growth rate of strain 26695 with that of other strains, following verification of the normality of the CFU variable through the Shapiro–Wilk test.

### Genetic sequencing and genomic analysis

After verification by gel electrophoresis, the PCR products were purified using the FastGene Gel/PCR Extraction kit (Nippon Genetics Co., Ltd, Tokyo, Japan). The extension product was prepared using the Big Dye Terminator v3.1 cycle sequencing kit (Thermo Fisher Scientific Inc., Waltham, MA, USA) through 30 cycles in a thermocycler programmed for 10 seconds of denaturation at 96°C, 5 seconds of annealing at 50°C, and 4 minutes of extension at 60°C. Subsequently, purification was carried out using the Performa DTR Gel Filtration Cartridges protocol (EdgeBio, San Jose, CA, USA) before Sanger sequencing on a SeqStudio platform (Thermo Fisher Scientific Inc.). The raw sequence outputs were initially processed using Finch software v.1.4.0 (49).

A bacterial clone (26695_Fr3-8) generated in this study underwent WGS to enable the assessment of the specific full-length genes for potential genomic rearrangements that may result from the mutagenesis experiments. DNA libraries were prepared using the TrueSeq DNA Nano kit (Illumina, Inc., San Diego, CA, USA) and were sequenced on the Miseq platform (Illumina Inc.). Raw data, obtained as short paired reads in fastq format, were quality-checked using FastQC v.0.11.9 (50). The sequences were then trimmed to remove low-quality bases (<Q30) and adapters using Trimmomatic v.032 (51). Sequences were thereafter *de novo*-assembled using SPAdes v.3.15.5 (52) under quality check using QUAST v.5.2 (53) before annotation with Prokka v.1.13.4 (54). Blast command lines (55) enabled the extraction of full-length *pbp1, pbp2 (fstI)*, *pbp3 (mrdA), FtsW, FtsX, FtsZ, LpoB, mreC, and MurJ* genes that would potentially affect AMX susceptibility. Sequence alignment to the reference genes from *H. pylori* 26695 (NC_000915.1) (43) and J99 (NC_000921.1) (21) was performed using MEGA software *v.10* and the MSA package in the R environment v.4.3.1 (47, 48, 56).

The core-genome alignment of strain *H. pylori* KIN76 and 20 reference strains representing different genetic populations of *H. pylori* (57) was obtained using panaroo (58) with a core-genome sample threshold of 0.95 and the aligner option set to “mafft.” A phylogenetic tree was inferred from the core-genome alignment by maximum likelihood using iqtree (59). The model “*GTR+F+I+R10*” was selected as the best-fit model (60). iTol web server was used to visualize the tree (61).

### Amoxicillin-PBP competitive binding assay with Bocillin

To assess the ability of PBPs of *H. pylori* to bind to AMX, we conducted competitive assays with Bocillin FL penicillin sodium salt (Thermo Fisher Scientific Inc.) (25). A previous protocol (26) was applied with some modifications, using *H. pylori* 26695 as the control strain. Bacterial cells (OD_590_ = 0.5–0.8) grown in 30 mL of brain heart infusion supplemented with 10% FBS were harvested by centrifugation and washed in D-phosphate-buffered saline (D-PBS). The cell pellet (corresponding to an OD_590_ of 20) was suspended in 1 mL of 200 mM Tris-Cl (pH 7.8)/20 mM EDTA/10 mg/mL lysozyme and incubated at 37°C for 30 minutes. A 100-µL sample was thereafter divided into eight tubes containing 1-mL D-PBS and centrifuged at 10,000 × *g* and 4°C for 15 minutes. Each of the eight resulting pellets was resuspended in 75 µL of 50 mM Tris-Cl (pH 7.8)/200 µM EDTA, and then, 25 µL of AMX was added at twofold serial concentrations ranging from 0 to 1.0 µg/mL diluted with distilled water. Reactions were incubated for 30 minutes at 37°C, washed with 1 mL prechilled D-PBS, and centrifuged for 10 minutes. Pellets were suspended again in 95 µL of 50 mM Tris-Cl (pH 7.8) and 5 µL of Bocillin at a 25 µM final concentration and then incubated in the dark at 4°C for 30 minutes. After washing in D-PBS and centrifugation, pelleted cells were resuspended in 50 mM Tris-Cl (pH 7.5)/0.5% SDS and incubated at 4°C for 10 minutes. The protein extracts were quantitated using the bicinchoninic acid assay protocol (Takara BCA, Protein Assay Kit, Takara Bio Inc.). Then, 24 µg of protein extracts was mixed with 5× SDS sample buffer, heat-denatured at 95°C for 5 minutes, and loaded into a Criterion TGX Precast Gel 7.5% (Bio-Rad Laboratories, Inc., Hercules, CA, USA) for separation by SDS-PAGE. The gel was visualized under the fluorescence Cy2 mode (for Bocillin-labeled proteins) and Cy5 mode (for the protein marker) using the ChemiDoc MP Imaging System (Bio-Rad), then soaked in a mixture of acetic acid and methanol for protein fixation and stained with the Coomassie Brillant Blue (CBB) method using QC Colloidal Coomassie dye (Bio-Rad). The CBB-stained gel was visualized under the CBB mode and used for the normalization of Bocillin fluorescence intensity in each lane according to the total protein intensity. Images were processed using Image Lab software v.6.1 (Bio-Rad) for band quantification. This experiment was independently conducted in triplicate, and results are presented as a bar chart showing the standard error, plotted in the R environment v.4.3.1 (47, 48).

### Protein structure modeling

To construct a 3D structure model of PBP1A, the amino acid sequence was processed through the Alphafold2 Colab platform (62) using the default parameters. From the resulting set of five models, the optimal performing model was selected based on the Molprobity scores (Tables S6 and S7) (63). The prediction of tunnels, pockets, catalytic residues, and binding sites was carried out using the Caver (64) and Cofactor (65) tools. The protein 3D structure was subsequently visualized through UCSF ChimeraX v.1.6.1 (66) and PyMOL v.2.5.4 (The PyMOL Molecular Graphics System, version 2.5.4, Schrödinger, LLC).

## Data Availability

The whole genome sequence of strain 26695_Fr3-8 has been deposited in the DDBJ/ENA/GenBank database under accession number JAXCGF000000000; the version described in this paper is version JAXCGF010000000. The *pbp1* sequence of donor strain KIN76 has been deposited in the GenBank database under accession number OR855674.

## SUPPLEMENTAL MATERIAL

File S1 msphere.00089-24-s0001.docx Supplemental tables and figures.

File S2 msphere.00089-24-s0002.xlsx Tables S6 to S17.

ASM does not own the copyrights to Supplemental Material that may be linked to, or accessed through, an article. The authors have granted ASM a non-exclusive, world-wide license to publish the Supplemental Material files. Please contact the corresponding author directly for reuse.
