# Supplementary material for: Synergistic effects of novel penicillin-binding protein 1A amino acid substitutions contribute to high-level amoxicillin resistance of Helicobacter pylori
Source: mSphere. 2024 Aug 1;9(8):e00089-24. doi: 10.1128/msphere.00089-24 (PMC11351044; doi:10.1128/msphere.00089-24)
Supplement: File S1 — Supplemental tables and figures. [file msphere.00089-24-s0001.docx]

**Synergistic effects of novel penicillin-binding protein 1A amino acid substitutions contribute to high-level amoxicillin resistance of *Helicobacter pylori***

**Supplementary material**

**Suppl. Table 1** Physicochemical properties of the tunnels of strain 26695 PBPs as estimated by SnapGene software *v.6.0*

| **Protein name** | **Encoding gene name** | **Number of amino acids** | **Molecular weight (kDa)** | **Isoelectric point (pI)** | **Charge at pH=7.0** |
| --- | --- | --- | --- | --- | --- |
| PBP1A | *pbp1* | 659 | 74.3 | 9.7 | 15.6 |
| PBP2 | *ftsI* | 615 | 69.4 | 9.82 | 21.6 |
| PBP3 | *mrdA* | 588 | 66.8 | 9.5 | 14.1 |

**Suppl. Table 2** Catalytic residue prediction by CAVER

| **Catalytic residue inside the pocket** | **Pocket score** | **Volume (A°)** | **Druggability** | **Residues** |
| --- | --- | --- | --- | --- |
| S368 | 100% | 735 | 0.13 | 415–417, 432–435, 541, 556–559, 561, 562, 593, 594 |

**Suppl. Table 3** Pocket prediction by CAVER

| Pocket ID | Relevance score (%) | Volume (A°) | Druggability |
| --- | --- | --- | --- |
| 1 | 100 | 735 | 0.13 |
| 2 | 99 | 1949 | 0.04 |
| 3 | 78 | 1087 | 0.38 |
| 4 | 59 | 673 | 0.22 |
| 5 | 50 | 697 | 0.02 |
| 6 | 49 | 963 | 0.06 |
| 7 | 46 | 394 | 0.11 |
| 8 | 46 | 459 | 0.01 |
| 9 | 46 | 745 | 0.05 |
| 10 | 43 | 646 | 0.31 |

The pocket with the highest relevance score was selected for tunnel prediction.

**Suppl. Table 4** Ligand binding site prediction by COFACTOR

| **Rank** | **CscoreLB** | **PDB hit** | **TM-score** | **RMSD** | **IDEN** | **Cov.** | **BS-score** | **Lig. name** | **Predicted binding site residues** |
| --- | --- | --- | --- | --- | --- | --- | --- | --- | --- |
| 1 | 0.22 | 2y2gA | 0.533 | 3.06 | 0.211 | 0.581 | 1.66 | NA | 433, 541, 556, 557, 558 |
| 2 | 0.21 | 2xd1A | 0.536 | 3.25 | 0.214 | 0.589 | 1.51 | CEF | 67, 368, 435, 470, 556, 557, 558, 559, 560 |
| 3 | 0.17 | 2olvA | 0.752 | 3.64 | 0.236 | 0.856 | 1.47 | MOE | 86, 124, 127, 128, 135, 139, 140, 143, 168, 203, 204, 205, 206, 207 |

*(a) CscoreLB stands for Confidence score of Ligand-Binding and is the confidence score of the predicted binding site. CscoreLB values range between [0–1]; where a higher score indicates a more reliable ligand-binding site prediction.*

*(b) BS-score stands for Binding Site score and is a measure of local similarity (sequence and structure) between the template binding site and the predicted binding site in the query structure. Based on large-scale benchmarking analysis, a BS-score >1 reflects a significant local match between the predicted and template binding sites.*

*(c) TM-score stands for Template Modeling score is a measure of global structural similarity between the query and template proteins.*

*(d) RMSD stands for Root Mean Square Deviation and corresponds to the RMSD between residues that are structurally aligned by TM-align.*

*(e) IDEN stands for Identity and corresponds to the percentage sequence identity in the structurally aligned region.*

*(f) Cov. or Coverage represents the coverage of global structural alignment and is equal to the number of structurally aligned residues divided by the length of the query protein.*

Rank 2 shows the prediction of the binding sites for cefotaxime (CEF), an antibiotic belonging to the beta-lactam family, which shares a similar lactam ring to amoxicillin.

**Suppl. Table 5** Tunnel prediction by CAVER

| **Id** | **Bottleneck radius (A°)** | **Length (A°)** | **Curvature** | **Throughput** | **Residues** |
| --- | --- | --- | --- | --- | --- |
| 1 | 2.5 | 1.4 | 1.0 | 0.95 | 368, 371, 416, 417, 432–435, 541, 555–558, 562, 593, 594, 595 |
| 2 | 2.1 | 8.7 | 1.1 | 0.84 | 365, 367, 368, 371, 414–417, 432–435, 467, 470–472, 541, 555–560, 562, 593–595 |
| 3 | 1.8 | 6.7 | 1.1 | 0.83 | 368, 371, 415–419, 432–435, 541, 555–558, 562, 593–595 |
| 4 | 1.0 | 30.5 | 1.5 | 0.31 | 365, 367, 368, 371, 396, 411, 413–417, 432–435, 438–444, 447, 450, 466–472, 541, 555–560, 562, 593–595 |

Note that both residues in positions 558 and 562, as well as those in positions 593 and 595, are shared among all the predicted tunnels.

**
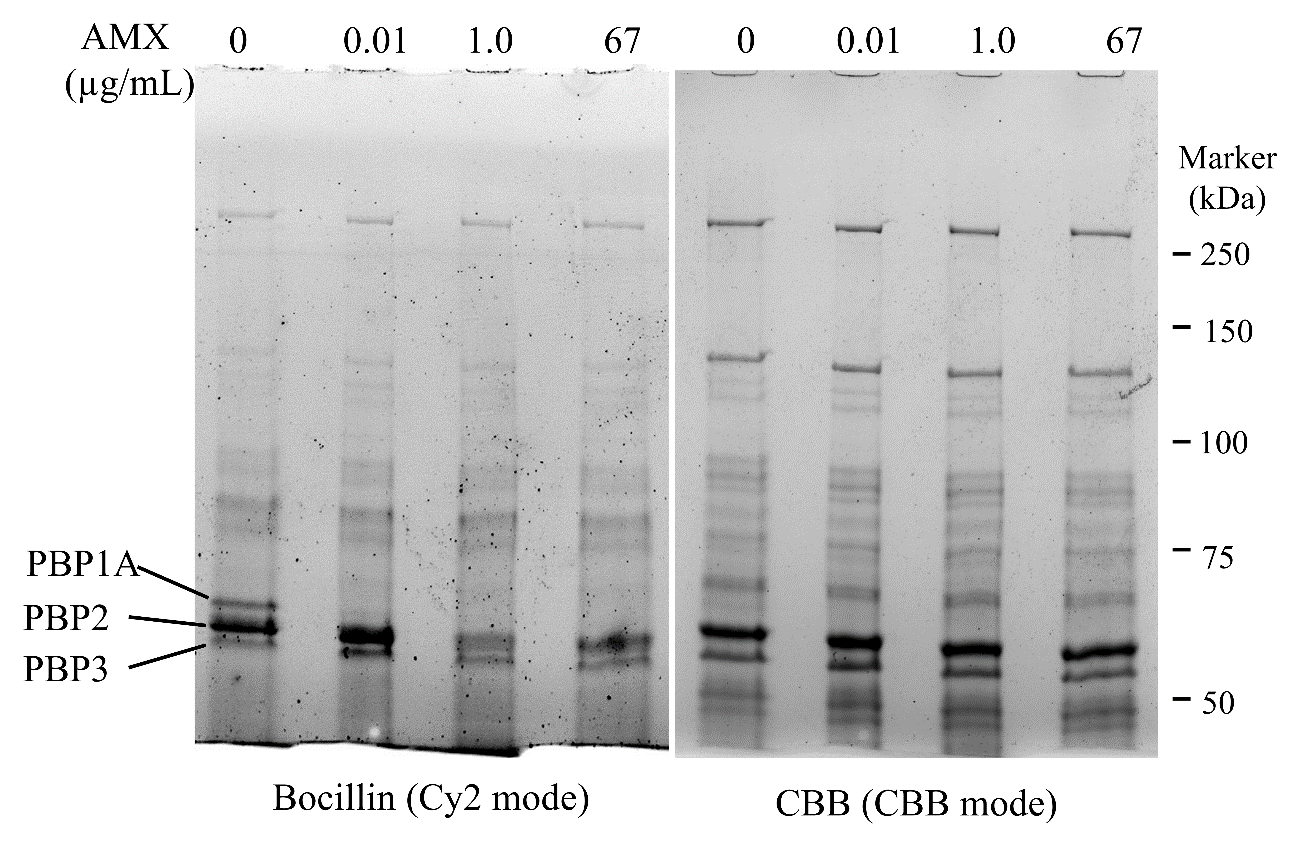
**

**Suppl. FIG 1** Protein bands detected by Bocillin with or without AMX pre-incubation. After SDS-PAGE, the Bocillin image (left gel) was captured in Cy2 mode. The bands corresponding to PBP1A, PBP2, and PBP3 are indicated in the 0 µg/mL AMX pre-incubation lane. The PBP2 resolved into two bands, and a total of four bands were distinguished by Bocillin in the lane corresponding to 0 µg/mL.

AMX, a phenomenon probably attributable to post-trans translational modifications described in

procaryotes (1, 2). Aside from the bands between 50 and 75 kDa, other faint bands are visible, referring to non-specifically labeled proteins by Bocillin or naturally fluorescent proteins, as confirmed by the electrophoresis of lysate without Bocillin labeling (data not shown). The total protein in each lane was shown after Coomassie Brilliant Blue (CBB) staining (right gel), representing the total protein loaded in each lane. The total protein intensity from each lane was used for Bocillin band normalization, as well as quantification in FIG 4C and 4D.


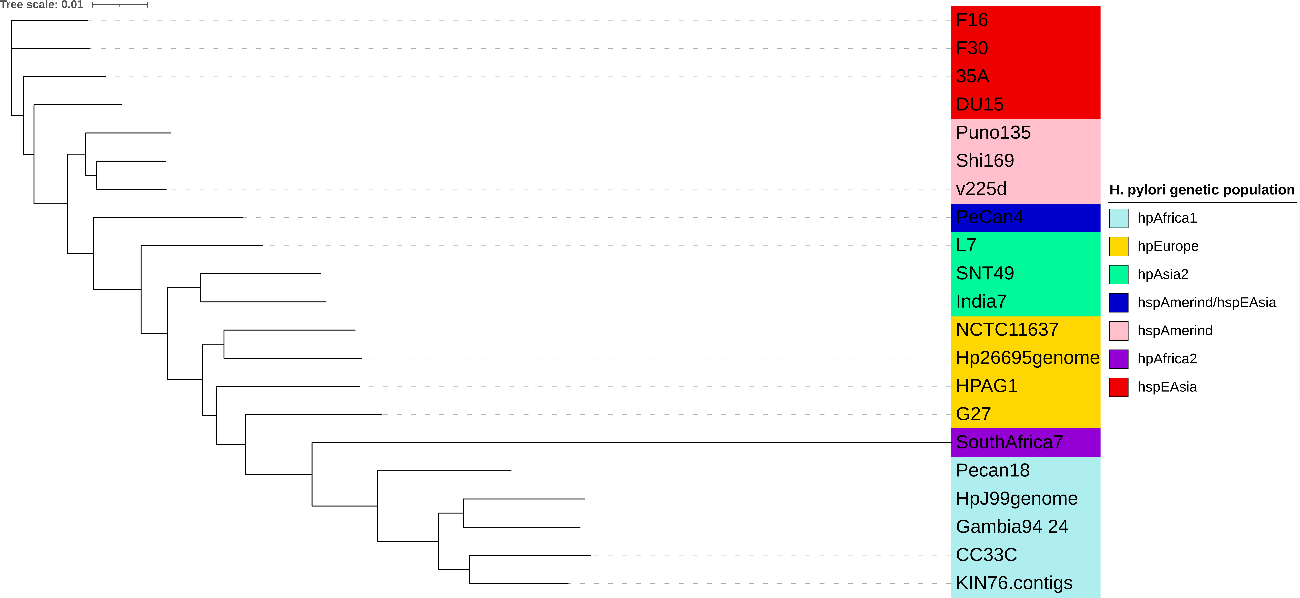


**Suppl. FIG** **2** Phylogenetic tree of H. pylori strains KIN76 and 26695

The phylogenetic tree was constructed from the core-genome alignment by maximum likelihood. Twenty *H. pylori* strains with previously assigned genetic populations, including *H. pylori* 26695, were used as references. Different *H. pylori* populations are indicated on the tree by different colors. Strain *H. pylori* KIN76 clustered with strains J99, CC33C, Gambia94/24, and Pecan18, previously assigned to the *hpAfrica1* population*.*

**
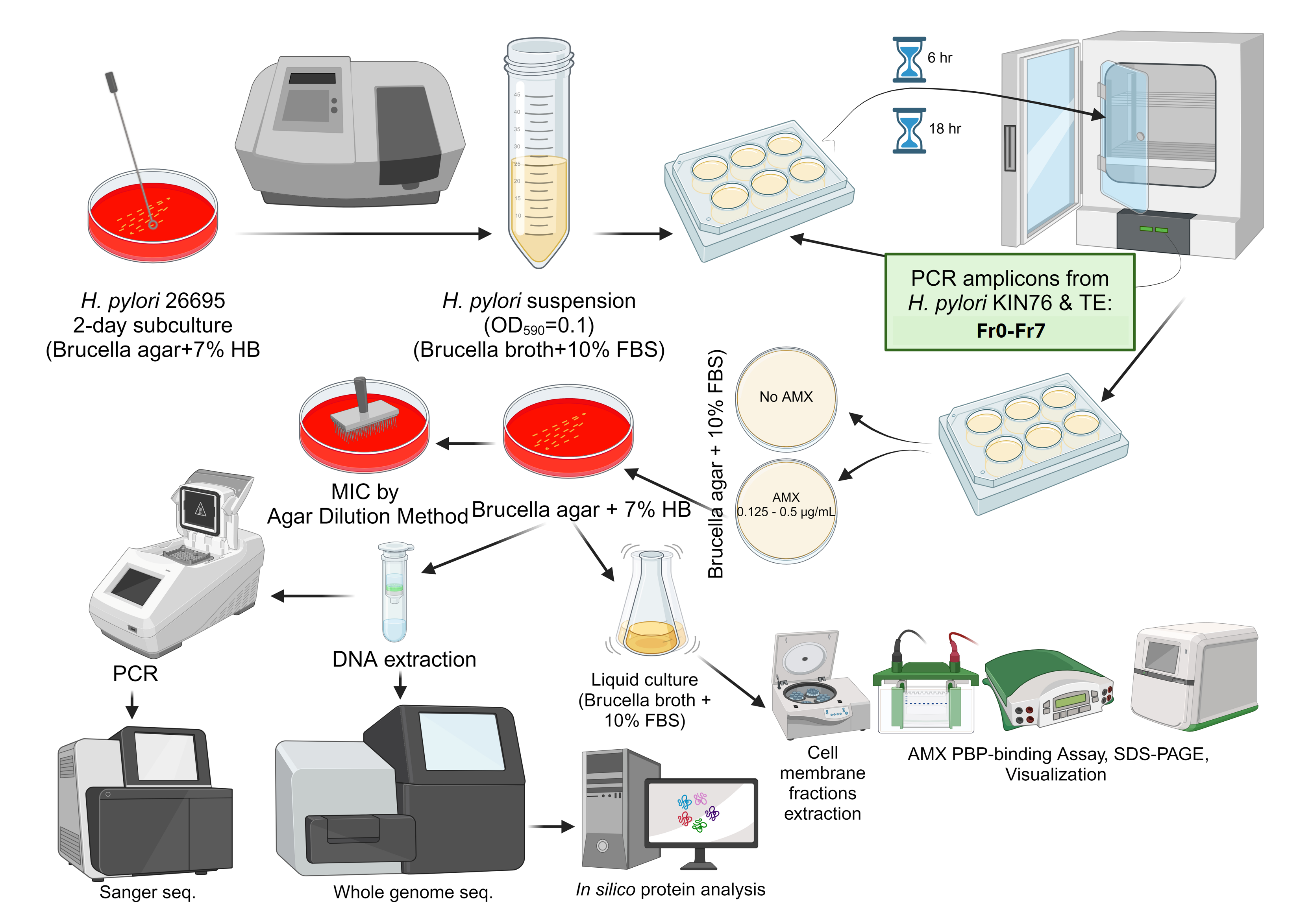
Suppl. FIG 3** Flowchart of the natural transformation experiment

A 2-day subculture of H. pylori strain 26695, the DNA recipient, was suspended in Brucella broth supplemented with 10% fetal bovine serum (FBS) adjusted to OD_590_=0.1, then divided into a 6-well plate (1 mL/well), which was incubated for 6 h under microaerophilic conditions. Next, 0.1 µg of the PCR-amplified *pbp1* gene fragments were added to the suspension; TE buffer (no DNA) as well as fragment Fr0 were used as negative controls. The culture was incubated for an additional 18 h under microaerophilic conditions and was thereafter suspended on selective Brucella agar plates supplemented with 10% FBS and containing AMX (0.5, 0.25, 0.125 µg/mL); an amoxicillin-free plate was used as a control. After 72 h incubation, eight colonies were picked from the selective plates and grown on AMX-free agar plates supplemented with 7% horse blood. This culture was used for DNA extraction followed by Sanger sequencing, AMX MIC determination using the agar dilution method, and a PBP binding assay.

**Supplementary references**

1. Macek B, Forchhammer K, Hardouin J, Weber-Ban E, Grangeasse C, Mijakovic I. 2019. Protein post-translational modifications in bacteria. Nat Rev Microbiol 17:651-664.

2. Rojo F, Berenguer J, Ayala JA, de Pedro MA. 1987. Variability in the posttranslational processing of penicillin-binding protein 1b among different strains of *Escherichia coli*. Biochem Cell Biol 65:62-7.
